# Risk factors of different hemoplasma species infections in cats

**DOI:** 10.1186/s12917-017-0953-3

**Published:** 2017-02-16

**Authors:** Michèle Bergmann, Theresa Englert, Bianca Stuetzer, Jennifer R. Hawley, Michael R. Lappin, Katrin Hartmann

**Affiliations:** 1 Clinic of Small Animal Medicine, Centre for Clinical Veterinary Medicine, LMU Munich, Veterinaerstrasse 13, 80539 Munich, Germany; 2 Center for Companion Animal Studies, Department of Clinical Sciences, College of Veterinary Medicine and Biomedical Sciences, Colorado State University, Fort Collins, CO USA

**Keywords:** Feline, Hemoplasmosis, *Mycoplasma* spp, PCR, Vector-borne, FeLV, FIV

## Abstract

**Background:**

Hemoplasma species (spp.) commonly cause infections in cats worldwide. However, data on risk factors for infections are limited. The aim of this study was to determine the prevalence of hemoplasma spp. infections in cats in Southern Germany and to assess risk factors associated with infection.

**Results:**

DNA was extracted from blood samples of 479 cats presented to different veterinary hospitals for various reasons. DNA of feline hemoplasmas was amplified by use of a previously reported PCR assay. Direct sequencing was used to confirm all purified amplicons and compared to hemoplasma sequences reported in GenBank. Results were evaluated in relation to the age, sex, housing conditions, feline leukemia virus (FeLV) and feline immunodeficiency virus (FIV) status of the cats.

The overall hemoplasma prevalence rate was 9.4% (45/479; 95% CI: 7.08–12.36). ‘*Candidatus* Mycoplasma (M.) haemominutum’ (Mhm) DNA was amplified from 42 samples, *M. haemofelis* from 2, and *M. haemocanis* from 1 sample. There was a significantly higher risk of hemoplasma infection in cats from multi-cat households, in outdoor cats, as well as in cats with FIVinfection and in cats with abortive FeLV infection, but not in cats with progressive or regressive FeLV infection.

**Conclusions:**

Mhm infection is common in cats in Southern Germany. Higher prevalence in multi-cat households and associations with FeLV infection likely reflect the potential for direct transmission amongst cats. Outdoor access, male gender, and FIV infection are additional risk factors that might relate to aggressive interactions and exposure to vectors.

## Background

Hemoplasma species (spp.) (or hemotrophic *Mycoplasma* spp.) are bacteria without cell walls that can cause hemolytic anemia in different species. At least 3 hemoplasma spp. have been described in cats, *Mycoplasma (M.) haemofelis* (Mhf), ‘*Candidatus (Ca.)* M. haemominutum’ (Mhm), and ‘*Ca.* M. turicensis’ (Mtc). Hemoplasma spp. attach to the external surface of red blood cells. Besides transmission through arthropod vectors, there is evidence of horizontal transmission (e.g., blood transmission during aggressive interactions between cats, blood transfusion) between cats [17].

Hemoplasma strains that occur in cats differ in their size and pathogenicity. Mhf and Mtc seem to have a higher pathogenicity and are more often associated with anemia than Mhm [2]. However, clinical relevance of hemoplasmas as a cause of anemia is not fully understood. Many authors believe them to be an important primary cause of anemia [20, 21, 25]; others regard hemoplasmas more as opportunistic pathogens [5, 12].

Natural hemoplasma infection is mostly subclinical. Clinical signs can occur after a longer latency under immunosuppressed conditions. Feline immunodeficiency virus (FIV) and feline leukemia virus (FeLV) infection can be associated with the development of clinical signs in some cats [4, 5, 24]. After developing clinical signs, alternating periods with anemia and subclinical phases can occur [2, 25].

Worldwide, cats are commonly infected by hemoplasma spp. [11, 12, 14, 21, 26, 27] and prevalence was found to be 9.9% in Switzerland [27] and 38.5% in Africa [14]. In Southern Germany, two studies investigated the prevalence of hemoplasma spp. infection in 135 cats [11] and in 296 cats [12] so far. These studies only evaluated preselected cats with anemia. Current data in a non-selected cat population are missing. Thus, the aim of the present study was to determine the prevalence of hemoplasma spp. by investigating blood samples of 479 cats by PCR. In addition, associations between hemoplasma spp. infections with the age, sex, housing conditions, and FIV and FeLV infection status of the cats were evaluated.

## Methods

### Animals

The 479 cats evaluated in this study were presented to different veterinary clinics in Southern Germany for various reasons. Health status of the cats was evaluated by physical examination. In each cat, a complete blood count (CBC) was performed and the FIV and FeLV status was examined. FIV antibodies were detected using a commercial enzyme-linked immunosorbent assay (ELISA) (SNAP Kombi Plus FeLV/FIV antibody test®, IDEXX GmbH, Ludwigsburg, Germany). FeLV infection status was investigated by performing tests for free FeLV p27 antigen using a commercial ELISA (SNAP Kombi Plus FeLV/FIV antibody test; IDEXX GmbH, Ludwigsburg, Germany), FeLV provirus using polymerase chain reaction (PCR) as well as anti-FeLV-p45 antibodies using an indirect ELISA, both as previously described [1]. Progressively FeLV- infected cats are persistently viremic and thus, FeLV antigen- and provirus-positive [9]. Regressively FeLV-infected cats are antigen-negative and provirus-positive; they are considered FeLV carriers. Cats with abortive FeLV infection never become viremic; they are antigen-, and provirus-negative but have FeLV-specific antibodies [9].

A total of 298 cats were male (62.2%) and 181 cats were female (37.8%) (Table 1). The cats´ ages ranged from 3 months to 19 years. Median age was 7.4 years (age of 40 cats was unknown). Of the 468 cats, 106 (22.6%) were purebred cats and 362 (77.4%) were Domestic Shorthair (DSH). Breed was unknown in 11 (2.3%) cats. Nine of 479 cats (1.9%) were progressively FeLV-infected, 7 of 479 cats (1.5%) were regressively FeLV-infected, and 22 of 479 cats (4.5%) were abortively FeLV-infected. A total of 7 cats were FIV antibody-positive (1.5%); two of these 7 FIV-infected cats were also progressively FeLV-infected.

**Table 1:** Cats with and without hemoplasma species (spp.) infection, and analysis of the risk factors housing conditions, feline immunodeficiency virus (FIV), and feline leukemia virus (FeLV) infection status

		*n*	Hemoplasma spp*.-*positive^a^	Hemoplasma spp*.-*negative^a^	*p*	Mhm^a^	Mhf^a^	Mhc^a^
Total	479		45 (9.4)	434 (90.6)	-	42 (93.3)	2 (4.5)	1 (2.2)
Age (median)		439	9.0 years	7.2 years	-	9.3	17	2
Gender	male	298	38 (12.8)	260 (87.2)	0.001	37 (97.4)	0 (0.0)	1 (2.6)
(*n* = 470)	female	181	7 (3.9)	174 (96.1)		5 (71.4)	2 (28.6)	0 (0.0)
Origin	shelter	56	10 (17.9)	56 (82.1)	0.104	10 (100.0)	0 (0.0)	0 (0.0)
(*n* = 479)	private	423	35 (8.3)	388 (91.7)		32(91.4)	2 (5.7)	1 (2.9)
Household	multi-cat	378	44 (11.6)	334 (88.4)	0.007	41 (93.2)	2 (4.5)	1 (2.3)
(*n* = 445)	single-cat	67	1 (1.5)	66 (98.5)		1 (100.0)	0 (0.0)	0 (0.0)
Access	outdoor	229	37 (16.2)	192 (83.8)	<0.0001	39 (95.1)	2 (4.9)	0 (0.0)
(*n* = 432)	indoor	203	4 (2.0)	199 (98.0)		3 (75.0)	0 (0.0)	1 (25.0)
FIV infection	positive	7	5 (71.4)	2 (28.6)	<0.0001	5 (100.0)	0 (0.0)	0 (0.0)
(*n* = 477)	negative	470	40 (8.5)	430 (91.5)		37 (92.5)	2 (5.0)	1 (2.5)
Progressive FeLV infection	positive	9	1 (11.1)	8 (88.9)	ns	1 (100.0)	0 (0.0)	0 (0.0)
(*n* = 479)	negative	470	44 (9.4)	426 (91.5)	ns	41 (93.2)	2 (4.5)	1 (2.3)
Regressive FeLV infection	positive	7	2 (28.6)	5 (71.4)	ns	2 (100.0)	0 (0.0)	0 (0.0)
(*n* = 479)	negative	472	43 (9.1)	429 (90.9)	ns	40 (93.0)	2 (4.7)	1 (2.3)
Abortive FeLV infection	positive	22	12 (54.5)	10 (45.5)	<0.0001	12 (100.0)	0 (0.0)	0 (0.0)
(*n* = 459)	negative	437	28 (6.4)	409 (93.6)		23 (82.2)	2 (7.2)	1 (3.6)

*n* numbers of cats in every group, *p p*-value, *ns* not significant, Mhm ‘*Candidatus* Mycoplasma (M.) haemominutum’, Mhf *M. haemofelis*, Mhc *M. haemocanis*; ^a^the percentages are presented in parenthesis

### Hemoplasma spp. PCR

Total DNA had previously been extracted from whole blood of the 479 cats using the MagNA Pure LC Total Nucleic Acid Isolation Kit (Roche Diagnostics AG, Rotkreuz, Switzerland) and stored at −80 °C until assayed in this study. The samples were thawed at room temperature and prepared for the amplification of hemotropic *Mycoplasma* spp. DNA in a previously reported conventional polymerase chain reaction (PCR) assay [10]. Briefly, each 2.5 μl DNA sample was added to the PCR master mix (10 mM Tris, pH 8.3, 50 mM KCl, 3.5 mM MgCl2, 200 μM each dNTP, 400 μM dUTP, 0.5 units uracil N-glycosylase (UNG) and 1.25 units Taq polymerase) with the final reaction volume of 25 μl.

PCR products were visualized on a 3% agarose gel using 6X EZVision One DNA Dye (Amersco; Solon, OH) according to manufacturer specifications.

Appropriate negative and positive controls were run with each sample. Positive PCR controls were obtained from diagnostic whole blood samples received by the Center for Companion Animal Studies (Department of Clinical Sciences, Colorado State University). Negative controls consisted of PCR (molecular grade) water being added in lieu of DNA template. Any positive sample was purified (QIAquick Gel Extraction Kit; Qiagen Germantown, MD) and sequenced to confirm genus and species (Colorado State University Proteomics and Metabolomics Facility; Fort Collins Colorado).

### Statistical analysis

Statistical analysis was performed with Graph PadPrism 6.0. Confidence intervals were determined by an exact binomial test. The exact binomial test was one-tailed and was used to prove the alternative hypotheses that the prevalence of hemoplasma infection was within the 95% confidence interval (CI). A significance level of < 0.05 was chosen. Fisher´s exact test was used to assess associations between hemoplasma infection, cats´ age, gender, and housing conditions, as well as FIV and FeLV status.

## Results

### Prevalence of hemoplasma spp. infections

The overall hemoplasma prevalence rate was 9.4% (45/479; 95% CI: 7.08–12.36) (Table 1). Mhm DNA was amplified from 42 samples, Mhf from 2 samples, and *Mycoplasma hemocanis* (Mhc) from 1 sample (Table 1). All PCR-negative and extract-negative controls were always negative in all assays.

### Hemoplasma-infected cats

Of the hemoplasma-positive cats, 84.5% (38/45) were male. Infected cats ages ranged from 9 months to 17 years (median 9 years). Most of the hemoplasma-infected cats were ≥ 9 years old (25/41); 8 cats were ≤ 3 years old (age of 4 cats was unknown).

Most of the infected cats were allowed to roam outside (90.2%; 37/41) and lived in multi-cat-households (97.8%; 44/45) (Table 1).

Ten of the infected cats were healthy and presented for neutering and/or routine health checks (22.2%; 10/45); the remaining cats were presented with a history of illness (77.8%; 35/45). Six infected cats had anemia (13.4%; 6/45), whereas 67 (21.1%; 67/317) of the non-infected cats had anemia. The hematocrit of the 6 hemoplasma-infected anemic cats ranged from 0.22 to 0.29 l/l (reference range: 0.30–0.44 l/l). None of these cats showed signs of regenerative anemia, such as polychromasia, macrocytosis, and in none of these cats nucleated red blood cells were present.

### Risk factors

Due to the low number of cats infected with the species Mhf or Mhc, all of the hemoplasma-infected cats were evaluated together for risk factor analysis. Male cats had a significantly higher risk of infection with a hemoplasma spp. than female cats (*p* = 0.001; odds-ratio (OR): 3.633; 95% CI: 1.586–8.322) (Table 1). In addition, cats with outdoor access (*p* < 0.0001; OR: 9.59; 95% CI: 3.35–27.42) and cats from multi-cat households (*p* = 0.007; OR: 8.66; 95% CI: 1.18–64.25) had a significantly higher risk of infection with a hemoplasma spp. Cats from shelters (*p* = 0.104; OR: 1.98; 95% CI: 92.8–421.9) had no significantly higher risk of hemoplasma-infection than cats from private homes.

Of the hemoplasma-positive cats, 5 cats were coinfected with FIV; 12 cats were abortively FeLV-coinfected, 2 cats were regressively and 1 cat was progressively coinfected with FeLV. Hemoplasma spp. bacteremia was significantly more often found in FIV-positive cats (*p* < 0.0001; OR: 26.88; 95% CI: 5.05–143.00) and in cats with abortive FeLV infection (*p* = 0.019; OR: 2.64; 95% CI: 1.26–5.51). Cats with progressive (*p* = 0.592) and regressive (*p* = 0.134) FeLV infections had no higher risk of being infected with hemoplasma spp..

### Mhc-infected cat

The Mhc-infected cat was a 10 months old, female neutered British Shorthair. The cat lived in a multi-cat household. The cat had no access to outdoors, no contact to dogs, and no travelling history. The cat was presented for routine health care. Physical and laboratory examination were unremarkable.

## Discussion

The present study revealed an overall prevalence rate of hemoplasma infections in cats of 9.4%. Worldwide, prevalence differs between about 10% in Switzerland [27] to over 30% in Africa [14]. Thus, prevalence of hemoplasma infection seems to be higher in countries with warmer climates. The infection rate in Southern Germany is similar to that in Switzerland, presumably due to the similar climate. An association between prevalence of potential vectors and prevalence of feline hemoplasma infections is likely [18, 23].

Similar to previous studies, Mhm was the most common hemoplasma spp. in the present study. None of the cats was positive for Mtc, although Mtc infections in cats from Southern Germany have been reported previously [11, 12]. Surprisingly, a 10 months old British Short Hair cat was infected with Mhc, the most prevalent hemoplasma spp. in dogs. Hemoplasmas are not necessarily strictly species-specific and there are reports stating that Mhc could infect cats and that cats might act as reservoir for Mhc [15, 16]. Mhc is most prevalent in dogs from Mediterranean countries; transmission is suspected *via Rhipicephalus sanguineus,* a tick that is not endemic in Germany [22, 28]. Source of Mhc infection in this cat of the present study is unclear. The cat was never allowed to go out and lived only indoors without any contact to dogs. Tick transmission therefore seemed unlikely; as this cat was still young, vertical transmission, which has been reported in cattle, might be the most likely source of infection [3].

Most of the Mhm- and Mhf-infected cats were older ( ≥9 years). Due to the subclinical nature of Mhm infection, this follows the presumption of becoming infected as a result of prolonged exposure during lifetime [23, 27].

As reported previously, infection was more prevalent in male cats and in cats with outdoor access [27]. This supports the hypotheses of horizontal transmission during aggressive interactions between cats and transmission through arthropod vectors [17]. In addition, cats from multicat-households were significantly more likely to be infected with hemoplasma spp., which could be due to a higher risk for aggressive interactions in multicat-environments.

Associations between hemoplasma spp. infection and clinical disease in naturally infected cats are not fully understood. Natural hemoplasma spp. infection is mainly subclinical in cats, but some cats develop severe anemia with fever, inappetence and lethargy [21]. It has been discussed whether retrovirus and hemoplasma infections are associated and whether immunosuppression could lead to enhanced pathogenicity of hemoplasma spp. [8, 23, 27].

A surprisingly strong association of FIV and hemoplasma spp. infection was found in the present study. Cats with hemoplasma infection were 26 times more likely to be FIV infected. This emphasizes, that hemoplasma spp. can be transmitted through similar routes as FIV, such as through biting. Similar to a recent study, FIV infection was predominantly associated with Mhm but not with Mhf and Mtc [23]. However, none of the hemoplasma FIV-coinfected cats showed signs of clinical manifestation of hemoplasmosis. It has been discussed that specific FIV strains might be the reason for more severe disease in coinfected cats [5], but this has never been proven so far.

In the present study, cats with hemoplasma infection were also more likely to be FeLV-infected. This is presumably attributable to outdoor access and multi-cat household, which increases the risk of FeLV infection through cat contact [7]. Only 3 of 28 FeLV hemoplasma-coinfected cats showed mild non-regenerative anemia which was likely caused by chronic kidney disease present in these 3 cats. In contrast, Harrus and colleagues found that Mhf-positive and cats positive for FeLV-p27 antigen were more often anemic than cats with Mhf infection alone [8]. However, most of the hemoplasma-positive cats in the present study were only abortively FeLV-infected (12/45), and cats with abortive FeLV infection never become viremic [9]. Abortive infections develop in immunocompetent cats. Due to effective immune response, these cats are able to terminate viral replication, and cats have no clinical signs [9]. Thus, immunocompetence is likely the reason for the missing enhanced pathogeniticity of hemoplasmas in the cats with abortive FeLV coinfections. In addition, all of the FeLV hemoplasma-coinfected cats were infected with Mhm, a strain that has an inherently low pathogenicity [2]. None of the progressively and regressively FeLV hemoplasma-coinfected cats showed clinical signs either. All this supports the hypothesis that the association of FIV and FeLV with hemoplasma spp. infection is likely the same mode of transmission and not necessarily an enhanced pathogenicity.

If hemoplasma spp. infection is clinically apparent, it usually results in regenerative hemolytic anemia [19]. In the present study, only 6 Mhm-infected of all 45 hemoplasma-infected cats had anemia. All of these cats had non-regenerative anemia, and other diseases that were likely responsible for the non-regenerative anemia (chronic kidney disease (*n* = 3), anemia of chronic disease caused by chronic respiratory tract disease (*n* = 2); tumor of the central nervous system (*n* = 1); chronic weight loss (*n* = 1)). Thus, other reasons than Mhm infection were presumably responsible for anemia in these cats. Although Mhf is thought to be more pathogenic, none of the Mhf-infected cats in the present study showed clinical signs. This could be due to a low hemoplasma load or infection with a less virulent strain. The Mhc-infected cat also showed no clinical signs of hemoplasmosis. Pathogenicity of this species in cats is unknown. Mhc can cause acute disease in immunosuppressed [6] or splenectomized dogs [13].

The major limitation of the study was the low number of cats with Mhf and Mhc infections and the fact that no cat in the present study was positive for Mtc. Thus, comparison between the different hemoplasma species was not possible. In addition, pretreatment of the cats in the present study was unknown, and treatment with antibiotics, e.g., doxycycline or enrofloxacin, prior to blood sampling could have led to termination of bacteremia and underestimation of hemoplasma prevalence [21].

## Conclusions

Feline hemoplasma infection is relatively common in cats, and if cats are infected, they are usually infected with Mhm, a species with low pathogenicity. A higher risk exists for male sex, outdoor access, multi-cat environment, and FIV- and FeLV-infected cats which seems to be related to similar mechanisms for transmission during aggressive interactions and through arthropod vectors.

## Abbreviations

Ca.: Candidatus
CBC: Complete blood count
DSH: Domestic Shorthair
ELISA: Enzyme-linked immunosorbent assay
FeLV: Feline leukemia virus
FIV: Feline immunodeficiency virus
M.: Mycoplasma
Mhc: Mycoplasma haemocanis
Mhf: Mycoplasma haemofelis
Mhm: ‘*Candidatus* Mycoplasma haemominutum’
Mtc: ‘*Candidatus* Mycoplasma turicensis’
n: Numbers of cats in every group
ns: Not significant
p: *P*-value
PCR: Polymerase chain reaction
spp.: Species
